# Supplementary material for: Multi‐dataset identification of innovative feature genes and molecular mechanisms in keratoconus
Source: J Cell Mol Med. 2024 Sep 19;28(18):e70079. doi: 10.1111/jcmm.70079 (PMC11412914; doi:10.1111/jcmm.70079)
Supplement: Supplementary file 2 — Material S2. [file JCMM-28-e70079-s002.zip › 7.GSEA analysis/2.ATOH7/KEGG_BASAL_CELL_CARCINOMA.html]

Details for gene set KEGG\_BASAL\_CELL\_CARCINOMA[GSEA]

|  || Dataset | ATOH7.group.cls#h\_versus\_l |
| Phenotype | group.cls#h\_versus\_l |
| Upregulated in class | h |
| GeneSet | KEGG\_BASAL\_CELL\_CARCINOMA |
| Enrichment Score (ES) | 0.61941147 |
| Normalized Enrichment Score (NES) | 1.8099613 |
| Nominal p-value | 0.0 |
| FDR q-value | 0.22996897 |
| FWER p-Value | 0.143 |
Table: GSEA Results Summary

  

Fig 1: Enrichment plot: KEGG\_BASAL\_CELL\_CARCINOMA      
 Profile of the Running ES Score & Positions of GeneSet Members on the Rank Ordered List

  

| SYMBOL | TITLE | RANK IN GENE LIST | RANK METRIC SCORE | RUNNING ES | CORE ENRICHMENT || 1 | WNT1 | na | 172 | 0.697 | 0.0671 | Yes |
| 2 | AXIN2 | na | 285 | 0.630 | 0.1290 | Yes |
| 3 | WNT9A | na | 551 | 0.534 | 0.1766 | Yes |
| 4 | LEF1 | na | 735 | 0.492 | 0.2222 | Yes |
| 5 | WNT6 | na | 825 | 0.474 | 0.2687 | Yes |
| 6 | SHH | na | 937 | 0.457 | 0.3128 | Yes |
| 7 | WNT5B | na | 1090 | 0.433 | 0.3532 | Yes |
| 8 | SMO | na | 2428 | 0.314 | 0.3471 | Yes |
| 9 | FZD7 | na | 2482 | 0.310 | 0.3777 | Yes |
| 10 | WNT9B | na | 2657 | 0.300 | 0.4037 | Yes |
| 11 | BMP4 | na | 2853 | 0.288 | 0.4279 | Yes |
| 12 | WNT11 | na | 3016 | 0.280 | 0.4521 | Yes |
| 13 | TCF7 | na | 3059 | 0.277 | 0.4796 | Yes |
| 14 | FZD10 | na | 3060 | 0.277 | 0.5083 | Yes |
| 15 | BMP2 | na | 3243 | 0.268 | 0.5307 | Yes |
| 16 | WNT7A | na | 3390 | 0.261 | 0.5535 | Yes |
| 17 | WNT8B | na | 3424 | 0.259 | 0.5793 | Yes |
| 18 | GLI1 | na | 4085 | 0.233 | 0.5844 | Yes |
| 19 | FZD2 | na | 4571 | 0.216 | 0.5927 | Yes |
| 20 | APC | na | 5634 | 0.182 | 0.5809 | Yes |
| 21 | WNT7B | na | 5905 | 0.174 | 0.5910 | Yes |
| 22 | GLI2 | na | 5995 | 0.171 | 0.6062 | Yes |
| 23 | WNT10A | na | 6547 | 0.156 | 0.6064 | Yes |
| 24 | TCF7L1 | na | 6644 | 0.153 | 0.6194 | Yes |
| 25 | WNT3A | na | 7555 | 0.129 | 0.6065 | No |
| 26 | FZD4 | na | 8076 | 0.117 | 0.6037 | No |
| 27 | WNT2 | na | 8209 | 0.114 | 0.6117 | No |
| 28 | DVL2 | na | 9335 | 0.093 | 0.5889 | No |
| 29 | APC2 | na | 10749 | 0.070 | 0.5554 | No |
| 30 | WNT4 | na | 10868 | 0.068 | 0.5590 | No |
| 31 | FZD8 | na | 11987 | 0.053 | 0.5322 | No |
| 32 | DVL3 | na | 12971 | 0.041 | 0.5081 | No |
| 33 | DVL1 | na | 13325 | 0.037 | 0.5017 | No |
| 34 | GLI3 | na | 14449 | 0.026 | 0.4720 | No |
| 35 | TP53 | na | 14698 | 0.024 | 0.4673 | No |
| 36 | WNT8A | na | 15047 | 0.021 | 0.4594 | No |
| 37 | STK36 | na | 15369 | 0.018 | 0.4520 | No |
| 38 | AXIN1 | na | 15654 | 0.015 | 0.4454 | No |
| 39 | GSK3B | na | 16028 | 0.013 | 0.4360 | No |
| 40 | FZD6 | na | 16592 | 0.008 | 0.4206 | No |
| 41 | SUFU | na | 17833 | -0.003 | 0.3851 | No |
| 42 | HHIP | na | 18105 | -0.005 | 0.3778 | No |
| 43 | CTNNB1 | na | 18214 | -0.006 | 0.3753 | No |
| 44 | FZD9 | na | 19998 | -0.022 | 0.3261 | No |
| 45 | WNT3 | na | 20041 | -0.022 | 0.3272 | No |
| 46 | FZD3 | na | 20400 | -0.026 | 0.3195 | No |
| 47 | FZD5 | na | 21276 | -0.035 | 0.2979 | No |
| 48 | TCF7L2 | na | 22134 | -0.044 | 0.2777 | No |
| 49 | FZD1 | na | 22280 | -0.046 | 0.2783 | No |
| 50 | PTCH2 | na | 24205 | -0.072 | 0.2303 | No |
| 51 | WNT5A | na | 24650 | -0.080 | 0.2258 | No |
| 52 | PTCH1 | na | 26724 | -0.119 | 0.1783 | No |
| 53 | WNT16 | na | 27162 | -0.130 | 0.1791 | No |
| 54 | WNT10B | na | 27303 | -0.133 | 0.1888 | No |
| 55 | WNT2B | na | 30926 | -0.244 | 0.1096 | No |
Table: GSEA details [plain text format]

  

Fig 2: KEGG\_BASAL\_CELL\_CARCINOMA      
 Blue-Pink O' Gram in the Space of the Analyzed GeneSet

  

Fig 3: KEGG\_BASAL\_CELL\_CARCINOMA: Random ES distribution      
 Gene set null distribution of ES for **KEGG\_BASAL\_CELL\_CARCINOMA**
